# Demographic supply-demand imbalance in industrial structure in the super-aged nation Japan

**DOI:** 10.1186/s12976-018-0091-z

**Published:** 2018-11-01

**Authors:** Naoki Kishida, Hiroshi Nishiura

**Affiliations:** 1 Graduate School of Medicine, Hokkaido University, Kita 15 Jo Nishi 7 Chome, Kita-ku, Sapporo-shi, Hokkaido 060-8638 Japan; 2 CREST, Japan Science and Technology Agency, Honcho 4-1-8, Kawaguchi, Saitama, 332-0012 Japan

**Keywords:** Population, Ageing, Mathematical model, Dependency ratio, Occupation

## Abstract

**Background:**

Japan has a rapidly decreasing population, with ultra-low fertility and extremely fast aging. The rapid dynamics constitute a warning that change in the industrial structure may be unable to meet the changing pace of age-dependent demand.

**Methods:**

The present study estimated the supply-demand imbalance by industrial sector, and we investigated the effectiveness of possible countermeasures. To quantify the demographic burden of different industry experts, we employed the dependency ratio to calculate the supply and demand of each industrial sector and occupation.

**Results:**

We identified an expected excess of demand in the health-care sector; the growth in that deficiency is likely to continue until 2045, when the elderly population is likely to reach a peak. By contrast, oversupply is expected in the education and construction sectors. An overall shortage of full-time workers is likely to continue until 2050, when we predict that Japan will lack 3.1–9.3 million full-time workers to satisfy the baseline demand level.

**Conclusions:**

Considering that the imbalance is evident over different sectors, interministerial regulation of occupational choice may need to be imposed, e.g., by drastically changing student sizes in different area of higher education. Japan may have to decide to downgrade its social services and potentially consider increasing immigrant workers.

**Electronic supplementary material:**

The online version of this article (10.1186/s12976-018-0091-z) contains supplementary material, which is available to authorized users.

## Background

The world population underwent an explosion in the last century, and that trend is expected to continue for another century [1]; however, the population has started to decrease in some industrialized nations. The impact on society of such time-dependent increases or decreases in population size is likely to be huge: the demographic dynamics directly affects the working-age population. Depending on the size and relative size of the working-age population, public systems need to undergo drastic change (e.g., with the pension system); education and training sectors likewise have to be updated in accordance with the expected demand based on demographic predictions [2–4].

**Fig. 1:**
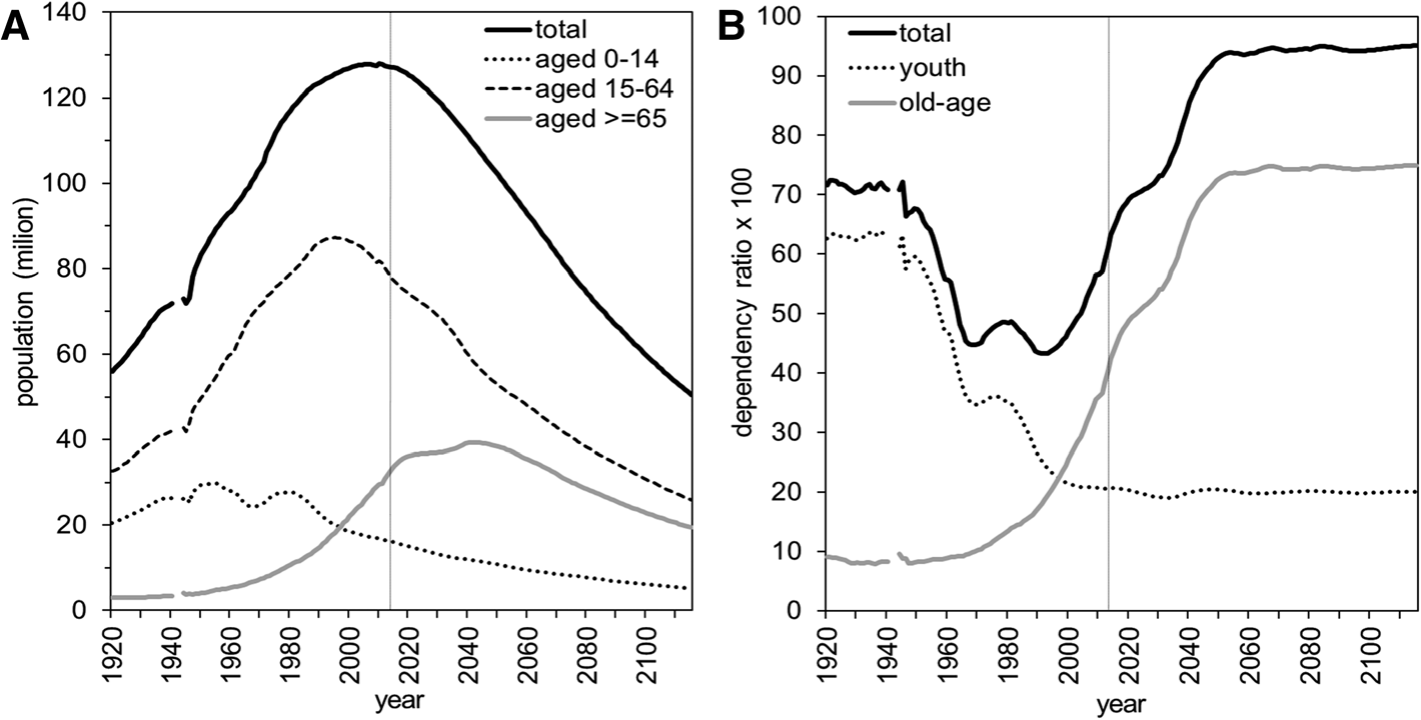
Age composition and dependency ratios in Japan for 1920–2115. **a** Age composition of the population according to three different groups. **b** Youth, old-age, and total dependency ratios calculated as the ratio of the number of, respectively, children (up to 14 years), elderly (65 years or over), and both of those dependent populations to the working-age population (15–64 years). The number of those dependents per 100 working adults is indicated. The vertical line shows 2017, when our analysis was conducted. The population data by 2015 is based on population estimates (*13*); the data thereafter are population projections (*14*). The age-specific population size was unavailable for 1941–43 because of World War II. For the same reason, Okinawa was excluded for 1945–46

Japan has experienced a successful, rapid demographic transition, with low birth and death rates [4, 5]; the second demographic transition was characterized, most importantly, by ultra-low fertility. In general, below replacement fertility is recognized as a core reason for aging. Also, changes in the country’s disease structure from infectious to chronic diseases should be noted, i.e., an epidemiological transition [6, 7]. Compared with other industrialized countries, the speed of transition in Japan has been extraordinarily rapid since the end of World War II (WWII); that country has faced a serious change in its age structure as a result of ultra-low fertility and extreme aging (Fig. 1) [8, 9]. In addition to changes in the age structure, the population has been decreasing since 2008 [8] (Additional file 1: Figure S1). The declining trend has not been unique to Japan; however, the extraordinary rapidity of its decline is a warning that the change in its industrial structure may be insufficient to keep pace with its developing age-dependent demands [10–14].

This situation demands that policies related to industrial development also need strong political guidance so that structural reforms comply with future social needs [15–18]. For example, in a rapidly decreasing population with ultra-low fertility, society needs to determine how many schoolteachers it should secure in 2040; with the massive increase in the number of elderly people, society has to evaluate how many helpers it needs to secure elderly care by 2035. To initiate debate on possible reformation of higher education sectors by occupational demand, it is vital to establish a rational basis for future human resource supply to be planned for each occupation [19–22].

The present study aimed to estimate and compare future supply and demand of human resources by occupation. We employed projected age-specific population size and labor force participation [13, 14, 23]; we calculated supply and demand by occupation and identified specific industries requiring drastic changes in the near future. We also examined possible countermeasures for addressing supply-demand imbalances by means of computational modeling.

## Methods

### Data source

Using age-dependent population estimates and projections for Japan [13, 14, 24], we calculated supply and demand. In addition to population data, we retrieved labor statistics from the Ministry of Internal Affairs and Communications [24–26], which classifies occupations by industrial sector. To calculate occupation-specific population size, we extracted the number of experts (i.e., by occupation) from additional data sources.

### Dependency ratio

Let *P*_a_(*t*) be the population size of age-group *a* in year *t* as extracted from the population census of 2015 and projection data for 2016–65. Adhering to convention, we hereafter consider the population in three age-groups: *a* = 1, representing the population aged 0–14 years; *a* = 2 aged 15–64 years; and *a* = 3, aged 65 years and older. The so-called old-age dependency ratio is calculated as *P*_3_(*t*)/*P*_2_(*t*); it represents the social burden of elderly people in year *t* from the viewpoint of an individual in the labor force. That ratio has been frequently used in economics and sociology to measure the pressure on the productive population.

Let *N*_i_(*t*) be the population size that an industry *i* would target as the recipient of a service. For instance, the service recipients of elementary schoolteachers would be children aged 7–12 years; those for kindergarten and nursery teachers would be children aged up to 6 years. In contrast, *N*_i_(*t*) of many health-care professionals would be elderly people, considering that over 70% of hospitalized patients are aged 65 years or older (except for specific expertise, such as pediatrics).

Given that labor statistics in Japan provide the industry-specific population size in industry *i*, *M*_i_(*t*), the industry-specific dependency ratio *r*_i_(*t*) is formulated as

1 $$ {r}_i(t)=\frac{N_i(t)}{M_i(t)} $$

which represents the pressure on an individual expert in industry *i*. The inverse of that quantity can be interpreted as the industry-specific support ratio, *s*_i_(*t*) [13].

### Supply-demand imbalance

In the future population, both *N*_i_(*t*) and *M*_i_(*t*) are expected to change owing to aging and decrease in the population size. Let *r*_i_ be the baseline of *r*_i_(*t*), which is measured as the average for 2002–06. We set 2002–06 as yielding the average level; we did so because we had access to a consistent statistical record of employed people with identical industrial classification from 2002. To achieve the service standard for the baseline period of 2002–06 in terms of manpower, while reflecting drastic changes in the service-recipient population *N*_i_(*t*), the expected demand for experts in industry *i* is calculated as

2 $$ {M}_{i, dem}(t)=\frac{N_i(t)}{r_i} $$

where *t* represents the future year. *M*_i,dem_(*t*) represents the required number of experts in industry *i* and year *t* to maintain the service level equivalent to the average of baseline years *t*_0_. Taking *M*_i,dem_(*t*_0_) as the baseline, we also calculate the relative change in the expert size, i.e., 1-*M*_i,dem_(*t*)/*M*_i,dem_(*t*_0_).

Subsequently, we estimate the total population size of workers in year *t*, *W*(*t*) to compute potential future supply. To do so, we used the percentage of labor force participation, defined as the proportion of the working population (see SI for detailed calculation). In Japan, 70 and 50%, respectively, of male and female working people aged over 15 years were categorized as having been engaged in employment as of 2015 [25, 26]. In recent years, those proportions have decreased with time. To predict the future labor force, we examined two possible scenarios (hereafter, referred to as the “labor scenario”) to describe the relationship between labor force participation and working-age population. In labor scenario 1, by linearly regressing labor force participation as a function of the proportion of the working-age population of 15–64 years, using the sum of least squares, we predicted future labor force participation. The scenario 1 assumes that no social change interferes the linear relationship between two variables. In labor scenario 2, we assume that the labor force participation in year *t*, *y*(*t*) is modeled as a nonlinear (exponential) function of the working-age population, *x*(*t*), i.e., *y*(*t*) = *a*exp(*bx*(*t*)) + *c* where *a*, *b* and *c* are parameters. Both *y*(*t*) and *x*(*t*) were dealt with as the proportion. The correlation assumes that, while there would be a physiological maximum age to which the working age can potentially be shifted, aging would have a negative impact on the labor force participation as a whole. The labor scenario 2 represents possible social adaptation to the decreasing population, e.g. naturally occurring delay in age of retirement and participation of homemakers in fulltime work. See SI for the detailed projection of the labor force population size.

Regarding industrial categories, supply is calculated by accounting for the change in *M*_i_(*t*). Again, we consider two different scenarios, i.e., migration scenario 1 assumes that no migration of workers takes place between industries, while migration scenario 2 assumes that workers of oversupplying industry have a probabilistic chance to seek for a different industry job in undersupplying industries, spending the delay of *τ* years for his/her adaptation. In migration scenario 1, we assumed that *M*_i_(*t*) changes proportionally to the total population size of workers, *W*(*t*) because the working-age population acts as the backup supply of human resources in industry *i*, i.e.,

3 $$ {M}_{i,\sup }(t)=W(t)\frac{M_{i, dem}\ \left({t}_0\right)}{\sum_j\ {M}_{j, dem}\ \left({t}_0\right)} $$

Assuming that society was satisfied with the granted fraction of industry *i*, the population in the baseline year *t*_0_, *M*_i,sup_(*t*) represents the possible (reduced) supply in future year *t* that could be maintained—even with the change in the size of the working-age population *W*(*t*). In migration scenario 2, workers of industry *i* move to other industries with a probability *α* if the industry *i* oversupplied *τ* years ago, i.e.,

4 $$ {M}_{i,\mathit{\sup}}(t)=\left\{\begin{array}{c}W(t)\frac{Mi, dem\left({t}_0\right)}{\sum \limits_j{M}_{j, dem}\left({t}_0\right)}+\alpha {\sum}_{k\in U}\left|{q}_{ik}\left(t-\tau \right)\right|\frac{q_i\left(t-\tau \right)}{\sum \limits_j{q}_j\left(t-\tau \right)},\kern1.00em \mathrm{if}\ {q}_i\left(t-\tau \right)\ge 0\\ {}W(t)\frac{Mi, dem\left({t}_0\right)}{\sum \limits_j{M}_{j, dem}\left({t}_0\right)}-\alpha \left|{q}_i\left(t-\tau \right)\right|,\kern1.00em \mathrm{if}\ {q}_i\left(t-\tau \right)<0\end{array}\right. $$

where *U* represents the group of industries with oversupply, and *q*_i_(*t*) is the imbalance in the size of experts in industry *i*, calculated by taking the difference between supply and demand:

5 $$ {q}_i(t)={M}_{i, dem}(t)-{M}_{i,\sup }(t) $$

If *q*_i_(*t*) is positive, demand is greater than supply in year *t*, indicating the need to produce more experts in the industrial area *i*. If it is negative, supply surpasses demand; those who expect a job in industry *i* are likely to miss the opportunity owing to the efflux of people. *q*_ik_(*t*) is migrated oversupply from oversupplied industry *k* to undersupplied industry *i* where there is an excess of demand. The redistribution is assumed to be proportional to the relative demand of industry *i* out of the total demand from all industries. The In our numerical illustrations using (4), we assumed *τ* = 5 years, accounting for the time-lag required for an adult to undertake a higher education in a different expertise area and varied *α* from 0 to 50% as part of sensitivity analysis.

Regardless of industrial specificity, the total volume of imbalance is calculated as

6 $$ u(t)={\sum}_i{M}_{i, dem}(t)-W(t) $$

for both labor scenarios 1 and 2.

We calculated *r*_i_(*t*) for 1949–2015. If the categorization of industry *i* changed over time, *r*_i_(*t*) was calculated for the available number of years with consistent recording of the specific industrial category. The baseline of *r*_i_(*t*) was the average over 5 years for 2002–06.

### Scenarios to address imbalance

To address the total imbalance *u*(*t*) in the labor scenario 1, we conducted an additional scenario analysis of possible interventions, potentially compensating for the shortfall in the future working population. By examining the classification of the working status of the population, we assessed possible scenarios for the following: (1) mobilizing homemakers for full-time work; and (2) extending the age of retirement. With the former, we considered hypothetical scenarios in which 20–100% of homemakers were recruited for employment. For the latter, we examined extending the retirement age by 10 years. The extension for 10 years is consistent with healthy life expectancy in Japan in the age of 70s, considering that the average life expectancy at birth is in early 80s, and is also roughly consistent with ongoing reform in various countries taking the retirement at life expectancy minus 15 years. We assumed workers aged 60–69 years would maintain the average labor force participation rate of workers aged 39–59 years. We did not consider these interventions for labor scenario 2, because the nonlinear relationship between working-age population and labor force participation could already reflect abovementioned changes in a natural manner.

### Summary of assumptions

For the above computations, we made the following assumptions:

1. The supply for 2002–06 was deemed optimal.
2. No migration took place from one industry to another in migration scenario 1, while inter-industry migration is allowed for a fixed proportion in migration scenario 2 if demand surpasses supply.
3. The quality of demand remained proportional to the population size of the service recipient.
4. Industry-specific supply was proportional to the relative size of industry-specific demand during the baseline years.
5. Labor force participation was proportional to the percentage of the working-age population in labor scenario 1, while we also accounted for societal adaptation to decreasing population in labor scenario 2 in which a nonlinear relationship was assumed between labor force participation and working-age population.

### Ethical considerations

The present study analyzed only secondary datasets that were publicly available, all of which were de-identified before collection. As such, ethical approval by the Institutional Review Board was not required for the present study.

## Results

**Fig. 2:**
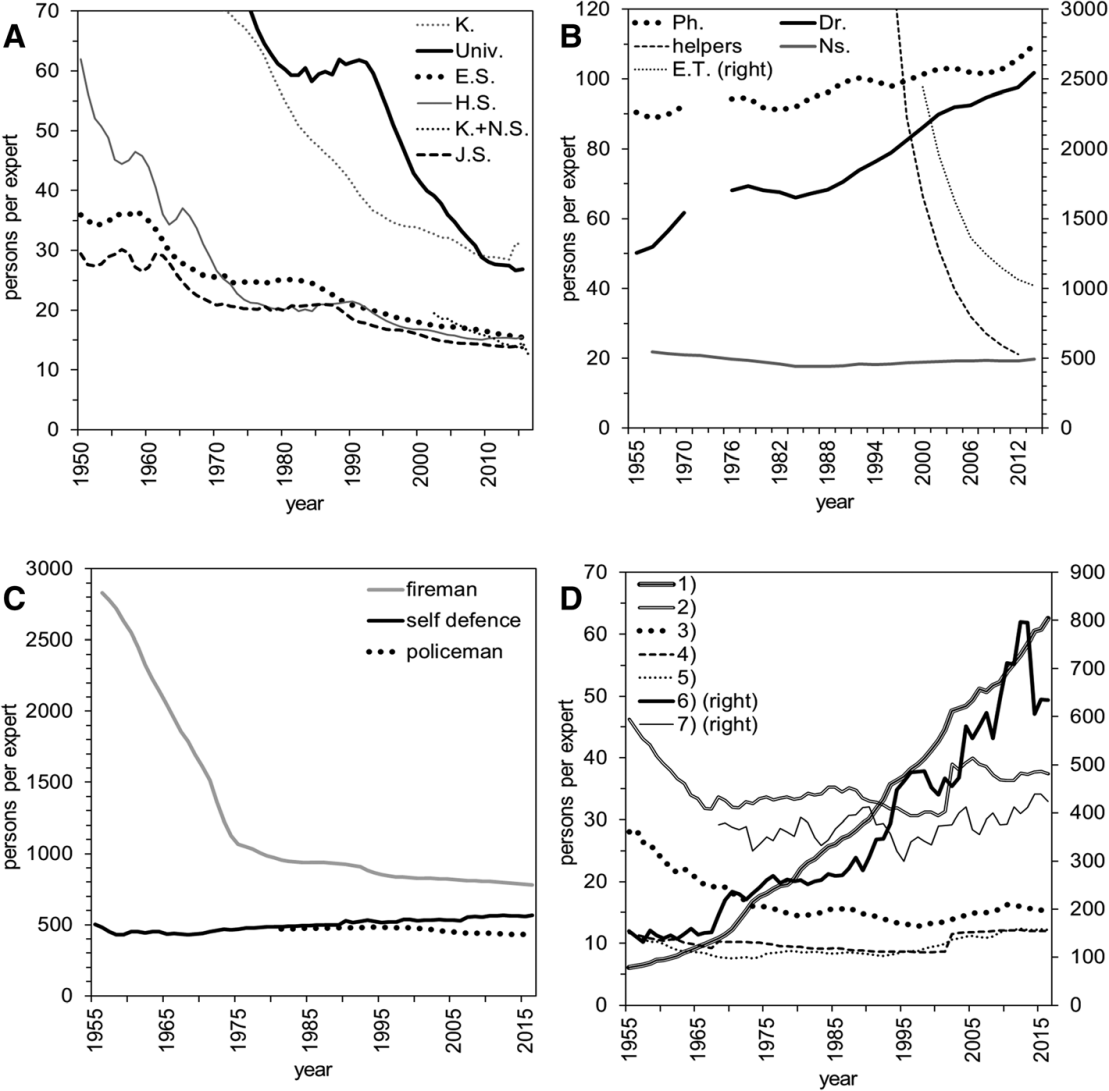
Estimated dependent persons per industry expert in Japan. **a** Education sector from 1950 to the present day. Statistical datasets for kindergartens plus nursery schools were available only from 2002. K., kindergarten; Univ., university; E.S., elementary school; H.S., high school; K. + N.S., kindergarten and nursery; J.S., junior high school. **b** Medical and health-care sector. Dr., physician; Ns., nurse; Ph., pharmacist; E.T., emergency medical technician. Physician and pharmacist data were available from 1955; nurse and helper data are from 1960 and 1990, respectively. Japan started licensing emergency medical technicians in 2000. Statistical data were recorded every 5 years from 1955 to 1970 but every 2 years since 1970. **c** Social security sector. The data for the Self-Defense Forces are from 1955, firefighters from 1956, and police officers from 1981. **d** Infrastructure sector from 1953. 1) Agriculture and forestry; 2) transport and communication; 3) construction; 4) wholesale and retail trade, eating and drinking places; 5) manufacturing; 6) fisheries (right); and 7) electricity, gas, heat supply, and water (right). Data for electricity, gas, heat supply, and water were available from 1968. The industrial categories were revised in 2002—especially for transport and communication and wholesale and retail trade, eating and drinking places. A category bearing the legend “right” indicates that the vertical axis is measured on the right vertical axis owing to a different scale of dependence compared with other closely related populations

We calculated the dependency ratio from the end of WWII to the present day for different job categories (Fig. 2). We grouped job categories into four sectors by industry: education; health care; social security; and infrastructure. These categories represent only a portion of all existing jobs, and only four categories with apparent temporal patterns are presented here. The service recipients in education and construction (that latter is one of the infrastructure sectors) are children and working-age people, respectively; the health-care service is mainly directed at the elderly. For most occupations, the estimated ratio of service recipients to providers has decreased or been constant since the end of WWII; however, an upward trend has been identified in health care and infrastructure. The industry-specific dependency ratio of Japan’s Self-Defense Forces has remained steady over time.

**Fig. 3:**
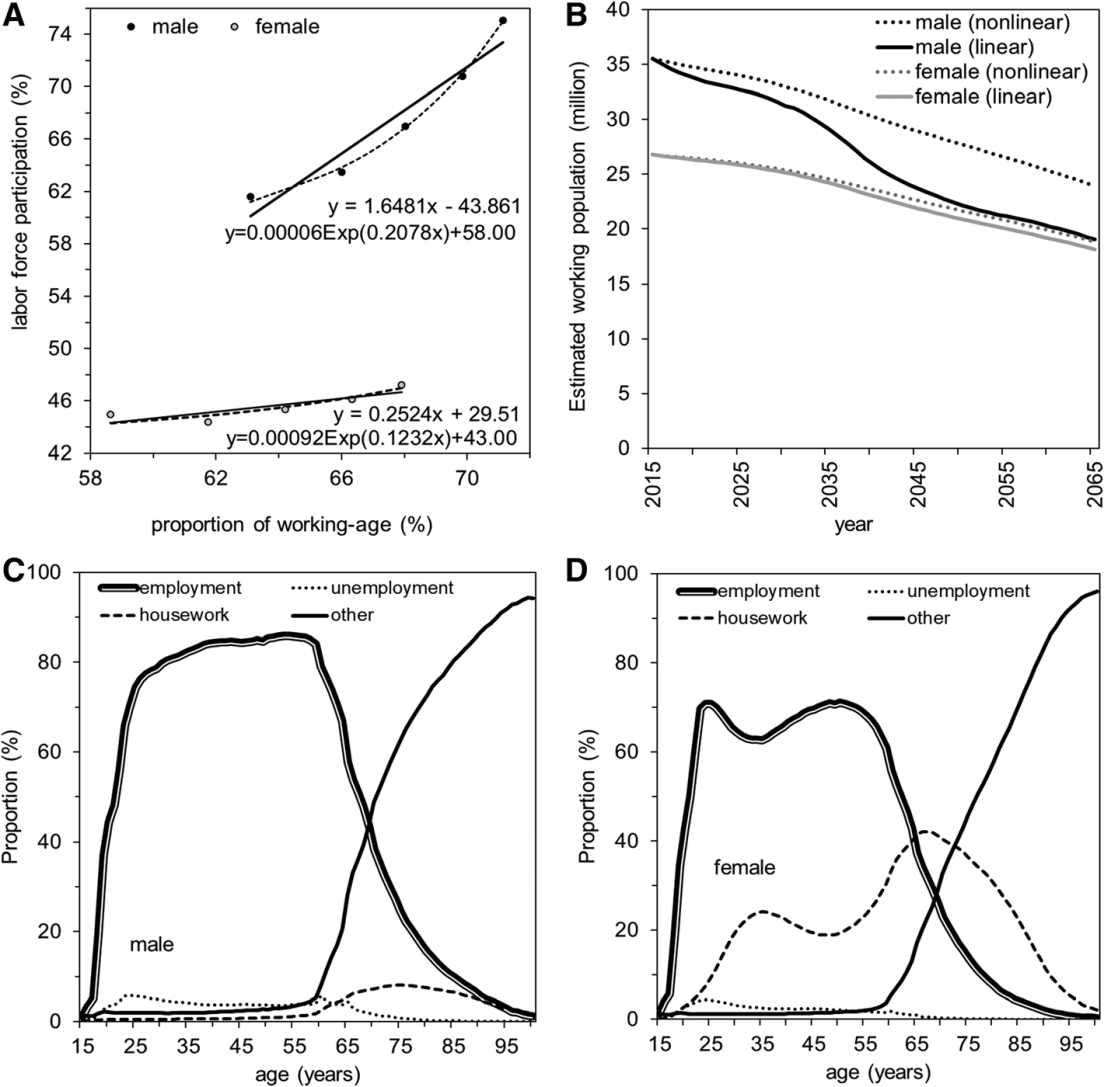
Predicted labor force and labor state in Japan. **a** Linear and nonlinear predictions of labor force participation using the working-age population size from 1995 (upper right for both males and females) and up to 2015 (lower left for both males and females). Best fit models along with expected value equations with estimated parameters are shown inside the panel. **b** Estimated number of workers based on linear and nonlinear extrapolations of labor force participation from 2015 to 2065. **c**, **d** Age-dependent snapshot of the labor state for males and females, respectively, according to the 2015 census

**Fig. 4:**
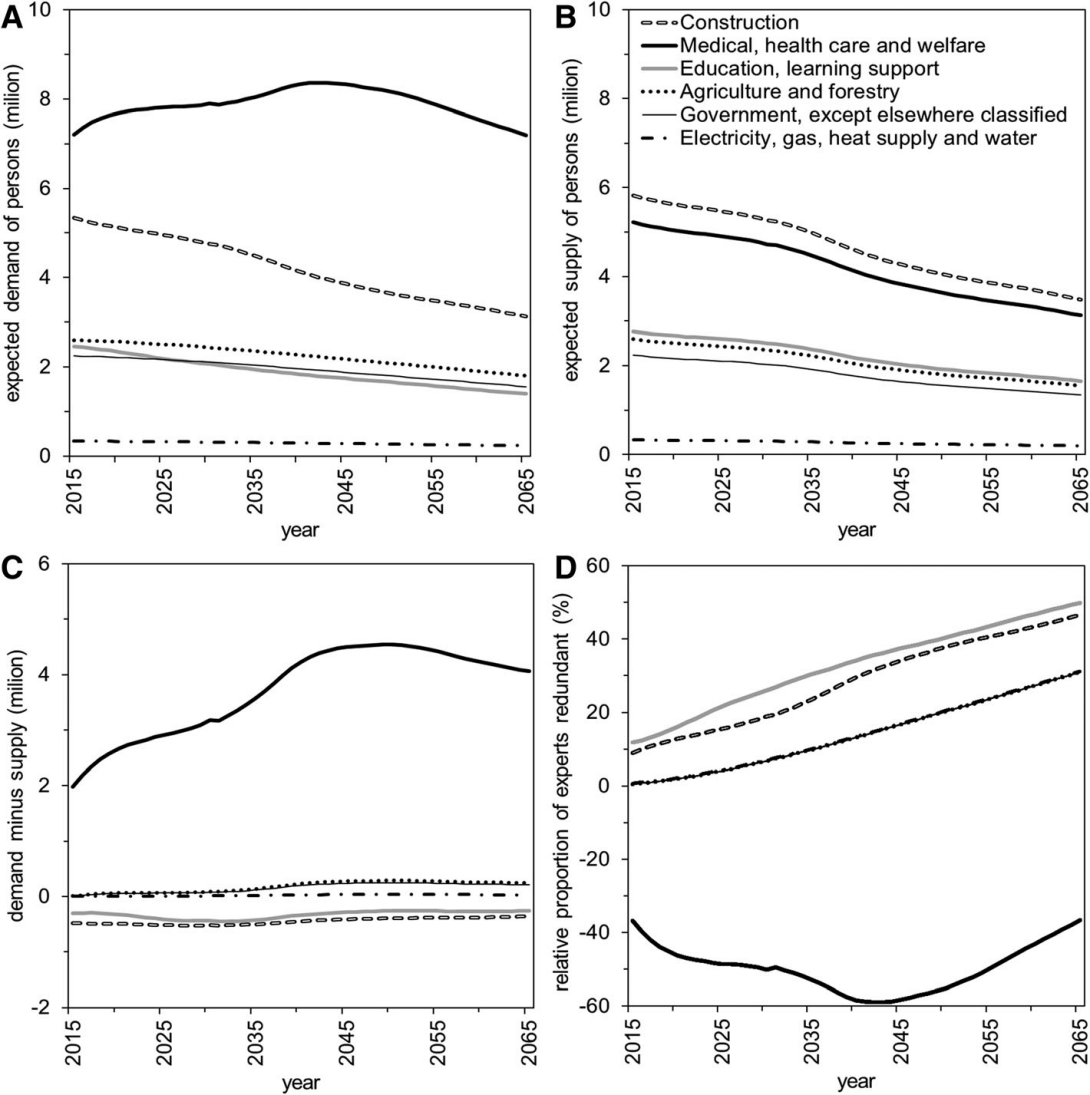
Predicted supply and demand of the labor force by industrial sector in Japan. The predicted supply and demand were computed by classification according to industrial sector using the average standard level for 2002–06. **a** Expected demand for industry-specific labor force to maintain the standard level. **b** Predicted supply of industry-specific labor force based on predicted worker size. Distribution of the industry-specific expert size was assumed to be maintained from that in 2015. **c** Predicted lack of experts, i.e., demand minus supply, and **d** relative proportion of redundant experts compared with the average for 2002–06

Assuming that the average dependency ratios from 2002 to 2006 would be maintained (as the baseline demand), we estimated the future demand, supply, and relative proportion of industry experts with respect to industry classification (Figs. 3 and 4). To predict the future working population, Fig. 3a shows linear and nonlinear extrapolations of labor force participation over 25 years using the proportion of working population by sex. The extrapolated labor force participation helps yield the forecasted volume of the future working population (Fig. 3b). Linear extrapolations perhaps represent a pessimistic scenario, predicting that male and female workers are only 24 and 22 million in 2045. However, nonlinear extrapolations reflect social adaptation to that change in a natural manner (e.g. naturally determined delay in the age of retirement), predicting that there will be 29 and 23 million male and female workers in 2045, respectively. Figure 3c and d shows the age-specific composition of the employment status in 2015, which we assumed to remain constant over time.

After selecting six different industrial categories, we made future predictions about demand (Fig. 4a) and supply (Fig. 4b). On the whole, demand forecasts decreased with time, reflecting the considerable decrease in the size of Japan’s population. Only demand in the health-care sector (i.e., the category encompassing medical, health-care, and welfare fields) was expected to increase up to around the year 2045 owing to aging (Fig. 4a).

Supply decreased in all industrial sectors. Supply and demand dynamically varied with time (Fig. 4c). Shortage in supply was evident for the health-care sector; demand was lower than supply in the education and construction sectors. The relative proportion of expert shortage was predicted to be positive compared with baseline for 2002–06 in all industrial categories other than health care (Fig. 4d). More precise predictions by occupational classification appear in Additional file 2: Figure S2. It is striking that all occupations in education will experience a monotonically increasing trend of redundancy; all occupations in the health-care sector will undergo a serious shortage after 2040 (Additional file 2: Figure S2). Given linear extrapolations, we found the overall imbalance between supply and demand to be as high as 3.3 million and 5.2 million workers in 2025 and 2035, respectively (Additional file 3: Figure S3). We expect this undersupply to escalate by 2050, with a maximum deficiency of 9.3 million workers. Nonlinear extrapolations indicated that the imbalance is eased compared with linear prediction, but qualitatively the overall declining trend with time was not different from that of linear extrapolations.

Predicted supply and supply-demand imbalance in the presence of inter-industry migration are shown by industrial sectors in Additional file 4: Figure S4 and Additional file 5: Figure S5. Within the parameter space that we explored, no drastic impact of inter-industry migration from oversupplying to undersupplying industries was observed.

**Fig. 5:**
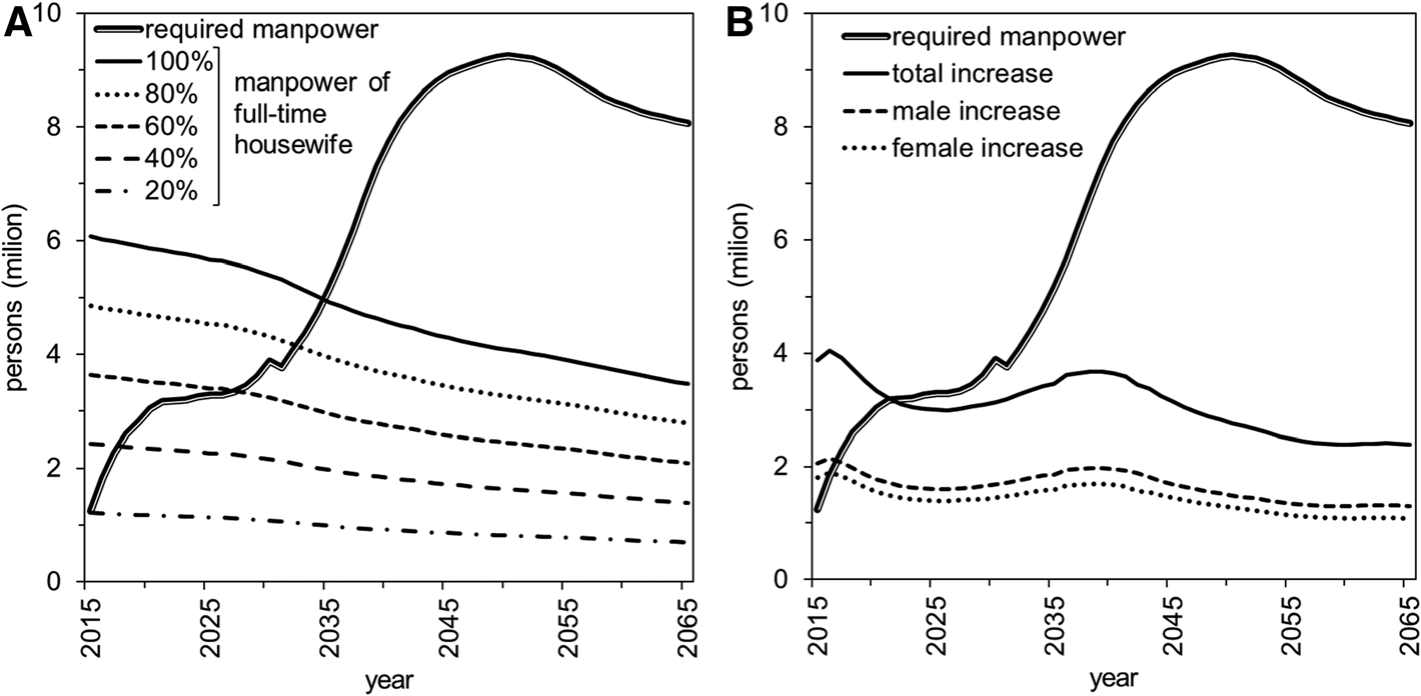
Predicted excess of overall demand and scenarios for recruiting additional full-time workers. In both panels, the thick solid line shows the estimated excess of demand in Japan; it is predicted to reach a maximum of over 9 million people by around 2050. That excess is compared with possible compensation scenarios. **a** Forecast of labor force replenishment by change in rate of full-time homemakers. The percentage represents the proportion of homemakers that could theoretically be recruited for full-time employment. The baseline (status quo) is 0 people. Even with 100% recruitment, the additional force cannot meet growing demand by 2035. **b** Forecast of labor force replenishment by 10-year delay in retirement age. The baseline (status quo) is that everyone is advised to retire at age 60 years and 0 people. The scenario is when that age is extended to 70 years. Demand exceeds the additional labor force by 2025

We conducted two scenario analyses to examine possible means of compensating for the discrepancy between supply and demand. Considering the large number of homemakers (Fig. 3d), they could hypothetically be recruited for additional work. Assuming that the age-specific distribution of women’s labor participation in 2015 continues in the future, Fig. 5a compares the size of the manpower shortage against the potentially recruitable size of additional manpower from homemakers. Even if all homemakers could be recruited, that policy alone cannot resolve the shortage by 2035.

Another—and perhaps more realistic—option is to extend the age of retirement. Assuming that the average labor force participation rates of males and females (estimated at 84.9 and 59.3%, respectively) were maintained during post-retirement ages of 60–69 years, an increase of about 4 million people could be expected (Fig. 5b). However, the shortage becomes apparent by 2022. Even if both options are combined, the shortage is not resolved by 2038 (Additional file 6: Figure S6).

## Discussion

Japan experienced a rapid economic recovery and population growth after WWII [8]. However, the demographic transition was extremely fast. Now, it is evident that the country is facing a rapid shift in age distribution and abrupt population decline [11, 19]. The present study demonstrates that such a demographic change will induce a considerable supply-demand imbalance in the industrial structure. We found that a large excess of demand in the health-care sector is to be anticipated; the growth of that deficiency is likely to continue until 2045, when the elderly population will probably reach a peak. By contrast, predictions indicate that the education and construction sectors will be oversupplied. An increase in the overall shortage of full-time workers should continue until 2050, by which time a deficiency of 9.3 million workers is anticipated. That shortfall cannot be easily addressed by recruiting homemakers to full-time work or extending the retirement age by 10 years.

Two important points should be stressed as take home messages from the present study. First, the future imbalance between supply and demand hinges on the industrial sector: more health-care personnel will have to be recruited and smaller numbers of teachers and construction workers will be demanded than actual supplies. Some studies have examined the macroeconomic aspect of this aging process using a different approach from the present investigation, and similar findings were indicated [9, 19–23]. Using a simpler approach with smaller number of assumptions, the present study examined such an extremely rapid aging and decline in population size in relation to the sustainability of Japan’s social services.

An important challenge toward correcting such imbalances in Japan’s current administrative system is independently regulating the number of industry experts handled by the various government ministries. For example, the Ministry of Health, Labour and Welfare is responsible for the shortage of physicians; however, the significant oversupply of schoolteachers is regulated by the Ministry of Education, Culture, Sports, Science and Technology. Ministerial independence may lead to maximization of a ministry’s benefit within its sector and protection of labor rights within that sector; but it does not necessarily comply with social demands. Thus, theoretical optimal allocation of human resources may not be deemed useful in the policy-making process—even within each ministry. As a possible idea, a top-down regulation of occupational choice might be imposed, e.g., by drastically adjusting the numbers of students within the various sectors of the higher education system. As another remark, considering that the fraction of children undertaking higher education at universities and colleges remains about a half of the population, paying an attention to less educated working population is also an important area to adjust the imbalance stated above.

The second point is that Japan faces a serious general shortage in its labor force as long as it aims to maintain the average dependency ratios of 2002–06. To correct that imbalance, either an increase in supply or a decrease in demand is requisite. With the former, we have demonstrated that the shortage can be partially addressed by responding as a society through recruiting additional people, including homemakers and retired individuals, to the labor force. Nevertheless, we have also shown that even with supplementation of the labor force, shortfalls will become apparent by 2022.

Thus, it is critical to consider the latter option, i.e., decreasing demand by changing the social system. It is possible to downgrade social services and emphasize that future social services will not be maintained at present levels. For example, geographically proximate communities may in the future use unified downsized administrative services. In addition, Japan currently anticipates that new technologies, including artificial intelligence, will help reduce the impact of social changes. Robotics and machine learning can reduce the need for humans to conduct various routine activities, which has already started in Japan, e.g. casher machines in supermarket, and also human resource demand in care-giving settings for elderly could drastically be eased. Innovation frequently occurs in unexpected areas, but that does not justify failure to undertake interventions, including downsizing.

Apart from demand reduction, it should be noted that there is a third political option, i.e., to increase the supply through immigration. We did not consider this option in the present study, because incorporating migrant workers would theoretically yield an infinitely large opportunity to compensate the undersupply of workers (and thus, the model would always indicate that importation of workers would resolve the ongoing problem). However, analysis of immigration, e.g. in healthcare sectors, requires various additional problems including language barrier, attractiveness in the Japanese working environment, and acceptance by elderly residents, and these aspects are subject for future studies.

Our findings would call for structural reform to sustain the future industries in Japan. To consider the industry-wide regulation, it must be remembered that the optimal allocation of human resources cannot be entirely addressed simply by a supply-demand analysis. Namely, we need to secure several professions that require an absolute number of workers. One example here is the Self-Defense Forces, which need to uphold current recruiting numbers to maintain national security. The absolute number of professional experts also has to be considered in the infrastructure sector, such as for handling water, gas, and electricity. Moreover, even a scientific demonstration of “optimal” allocation is not universally optimal in every single aspect, and thus, in reality, we must remember that theoretical optimal in a single scientific study is not a universal criterion. Another issue is the need for society to adjust to the changing working population [27, 28]. For example, to recruit elderly people into the labor force, future society has to ensure that they can be comfortably engaged in work beyond the perceived retirement age. In this way, quantitative allocation of specialty involves a number of challenges in its implementation, but at least the present study indicates qualitatively that the reformation of higher education structure is specifically called for.

The present study is not free from limitations. First, we set the average for 2002–06 to constitute a standard level for social services; but that choice was somewhat arbitrary. Qualitative findings would not have been affected if we had compared future situations against some period in the past. Second, we ignored mobility among different industrial sectors in migration scenario 1; however, depending on future change, we might expect a natural adjustment of imbalance owing to job-seeking behaviors. Whereas we partly addressed the issue of migration in migration scenario 2, our projections in migration scenario 1 may be regarded as a pessimistic scenario. Third, published studies have explicitly accounted for the future level of technology [19]. Rather than explicitly accounting for that aspect, we have shown that the issue can be comprehensively examined by simply extending the concept of the dependency ratio. Fourth, our analysis rests on simple ratios; it borrows the idea of the demographic dependency ratio. The problem of industry-specific population size may be resolved by addressing the imbalance we have presented; however, it should be remembered that aging occurs within the same working population group. Within-group aging is another problem for some occupations, including the Self-Defense Forces, police officers, and firefighters.

The overall decline in population size is not unique to Japan. But our analysis has shown that the rapidity of changing age and size distributions is exceptional; social change may be unable to match the demographic change. This study underscores the need to strategically develop concrete plans to regulate occupational choices in higher education (colleges and universities) as well as other countermeasures in a population-wide manner to avoid ministerial independent measures. We believe that our study has sufficiently characterized a critical basis on which systematic policies can be planned.

## Conclusions

The present study estimated the supply-demand imbalance by industrial sector, and we investigated the effectiveness of possible countermeasures. To quantify the demographic burden of different industry experts, we employed the dependency ratio to calculate the supply and demand of each industrial sector and occupation. Considering that the imbalance is evident over different sectors, interministerial regulation of occupational choice may need to be imposed, e.g., by drastically changing student sizes in different area of higher education. Japan may have to decide to downgrade its social services and potentially consider increasing immigrant workers.

## Additional files

Additional file 1: **Figure S1.** Relative decrease in the Japanese population for 2015–2115. The relative decrease in the population is measured for three age-groups and the whole of Japan. The age-groups are children (up to 14 years), working-age population (15–64 years), and elderly (65 years and over). (TIF 465 kb)

Additional file 2: **Figure S2.** Labor force imbalance between supply and demand by occupation (education, health-care, and social security sectors). Demand appears in the top three panels and supply in the middle panels. Demand minus supply is calculated as the relative redundancy compared with the standard average for 2002–06 (i.e., 0% for the average baseline value) in the bottom panels. Positive values indicate an excess of industry experts. **A** Education sector; **B** health-care sector; and **C** social security sector. **A** K. + N.S., kindergarten plus nursery; E.S., elementary school; J.S., junior high school; H.S., high school; Univ., university. **B** Dr., physician; Ph., pharmacist; E.T., emergency medical technician; Ns., nurse. (TIF 1116 kb)

Additional file 3: **Figure S3.** Overall demand-supply imbalance in Japan for 2015–65. Demand, supply, and their difference are presented for the whole of Japan. Because we considered two different labor scenarios (i.e., linear and nonlinear extrapolations of the future labor force), there are two possible results for supply and demand-minus-supply, respectively. (TIF 578 kb)

Additional file 4: **Figure S4.** Predicted supply of the labor force by industrial sector in Japan, accounting for inter-industry migration. The predicted supply was computed by classification according to industrial sector using the average standard level for 2002–06. Predicted supply of industry-specific labor force is shown based on predicted worker size, assuming that the fraction of inter-industry migrant from one industry to the other was (A) 0%, (B) 30% and (C) 50%, respectively. (TIF 466 kb)

Additional file 5: **Figure S5.** Predicted supply-demand imbalance of the labor force by industrial sector in Japan, accounting for inter-industry migration. The predicted supply and demand were computed by classification according to industrial sector using the average standard level for 2002–06. Predicted supply-demand imbalance of industry-specific labor force is shown based on predicted worker size, assuming that the fraction of inter-industry migrant from one industry to the other was (A) 0%, (B) 30% and (C) 50%, respectively. Positive value reflects the predicted shortage of experts, i.e., demand minus supply. (TIF 388 kb)

Additional file 6: **Figure S6.** Scenario analysis of the comparison between the expected excess of demand against possible additional number of workers. Deficiency of full-time workers (thick solid line) compared with possible countermeasure scenarios of recruiting 60% of homemakers, extending the retirement age by 10 years, and both options. Even if both countermeasures were undertaken, the shortfall would not be met by 2040. (TIF 568 kb)

## Abbreviation

WWII: World War II
