# Supplementary figures and images for: Demographic supply-demand imbalance in industrial structure in the super-aged nation Japan
Source: Theor Biol Med Model. 2018 Nov 1;15:19. doi: 10.1186/s12976-018-0091-z (PMC6211503; doi:10.1186/s12976-018-0091-z)

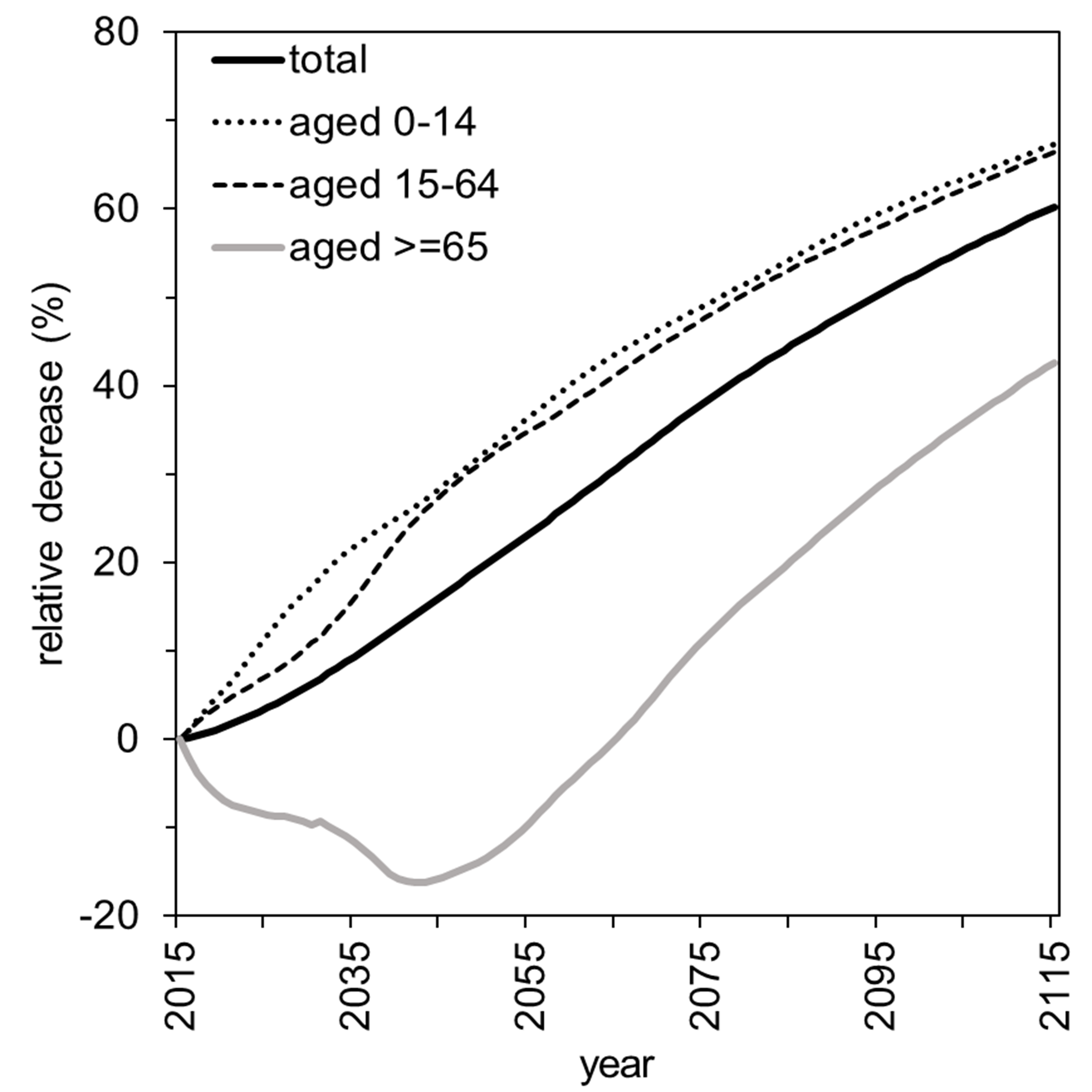

Supplement: Supplementary file 1 — Figure S1. Relative decrease in the Japanese population for 2015–2115. The relative decrease in the population is measured for three age-groups and the whole of Japan. The age-groups are children (up to 14 years), working-age population (15–64 years), and elderly (65 years and over). (TIF 465 kb) [file 12976_2018_91_MOESM1_ESM.tif]

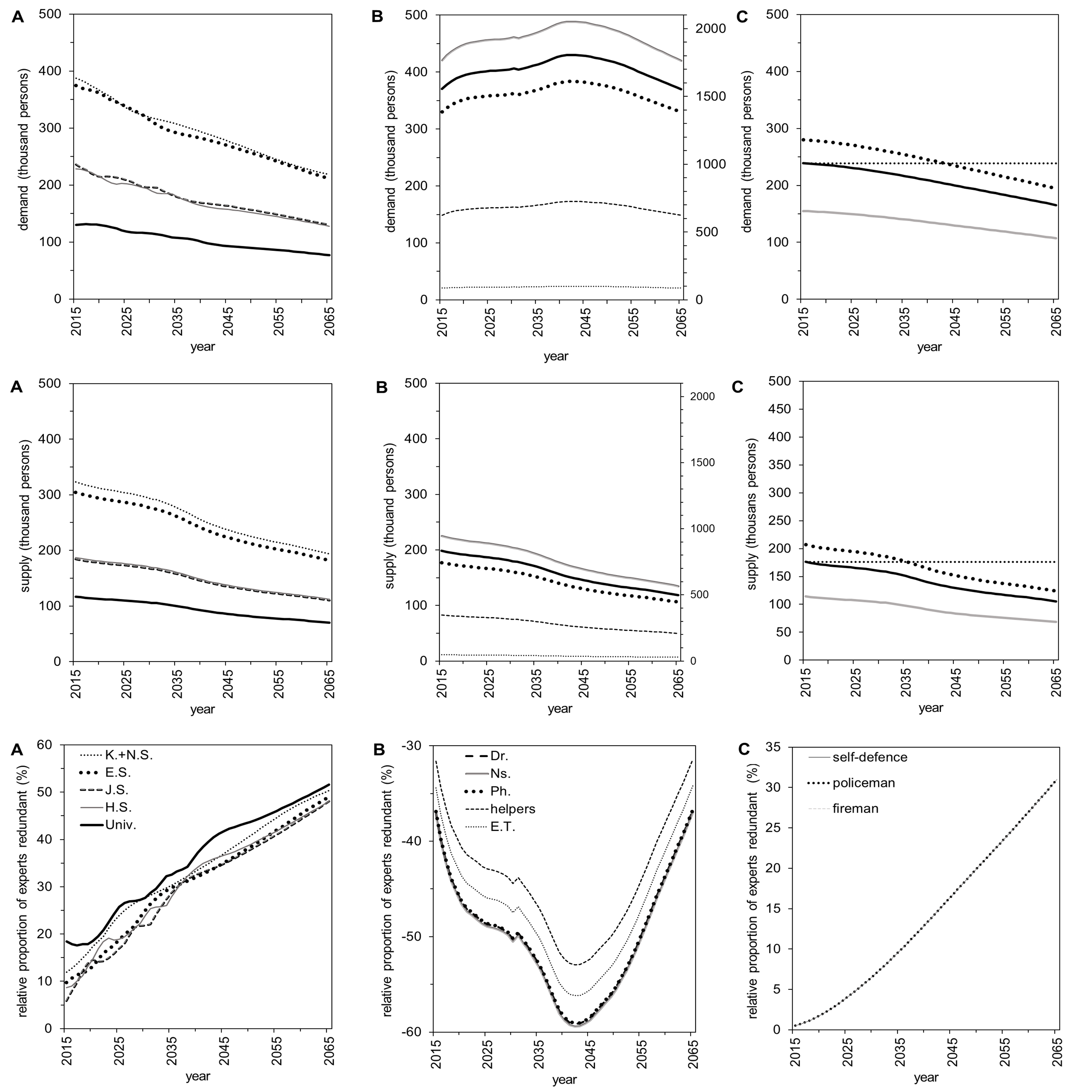

Supplement: Supplementary file 2 — Figure S2. Labor force imbalance between supply and demand by occupation (education, health-care, and social security sectors). Demand appears in the top three panels and supply in the middle panels. Demand minus supply is calculated as the relative redundancy compared with the standard average for 2002–06 (i.e., 0% for the average baseline value) in the bottom panels. Positive values indicate an excess of industry experts. A Education sector; B health-care sector; and C social security sector. A K. + N.S., kindergarten plus nursery; E.S., elementary school; J.S., junior high school; H.S., high school; Univ., university. B Dr., physician; Ph., pharmacist; E.T., emergency medical technician; Ns., nurse. (TIF 1116 kb) [file 12976_2018_91_MOESM2_ESM.tif]

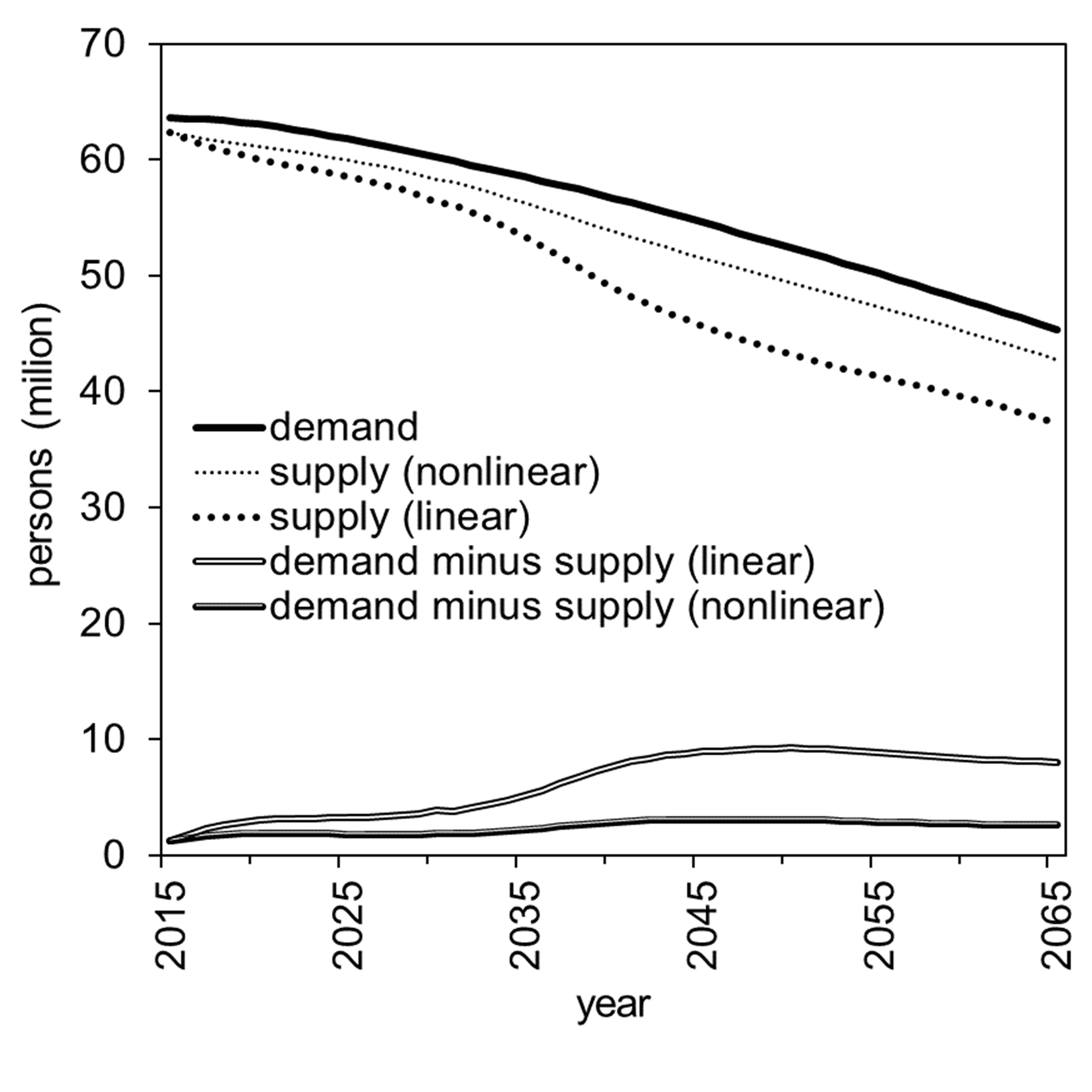

Supplement: Supplementary file 3 — Figure S3. Overall demand-supply imbalance in Japan for 2015–65. Demand, supply, and their difference are presented for the whole of Japan. Because we considered two different labor scenarios (i.e., linear and nonlinear extrapolations of the future labor force), there are two possible results for supply and demand-minus-supply, respectively. (TIF 578 kb) [file 12976_2018_91_MOESM3_ESM.tif]

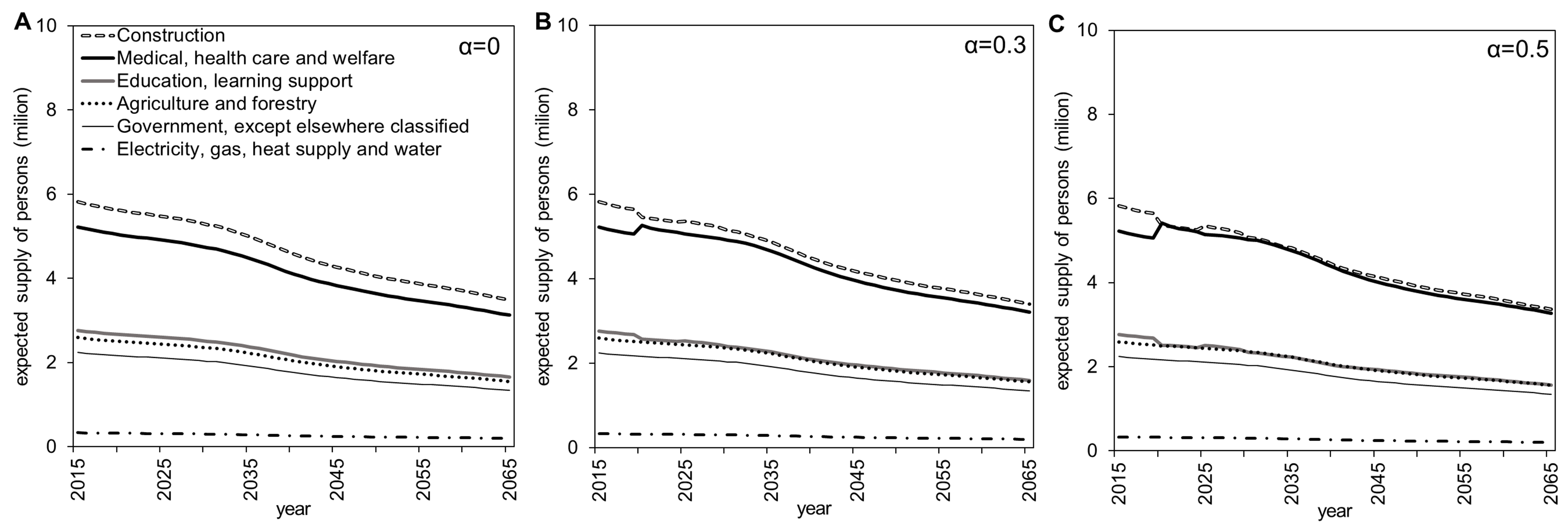

Supplement: Supplementary file 4 — Figure S4. Predicted supply of the labor force by industrial sector in Japan, accounting for inter-industry migration. The predicted supply was computed by classification according to industrial sector using the average standard level for 2002–06. Predicted supply of industry-specific labor force is shown based on predicted worker size, assuming that the fraction of inter-industry migrant from one industry to the other was (A) 0%, (B) 30% and (C) 50%, respectively. (TIF 466 kb) [file 12976_2018_91_MOESM4_ESM.tif]

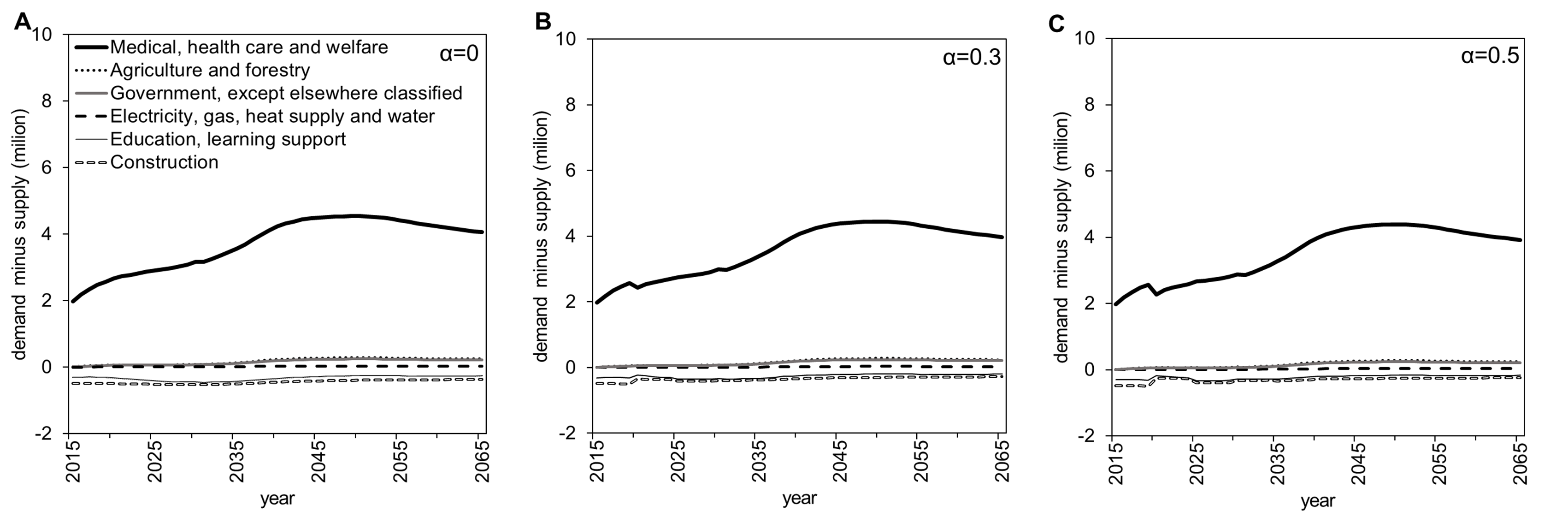

Supplement: Supplementary file 5 — Figure S5. Predicted supply-demand imbalance of the labor force by industrial sector in Japan, accounting for inter-industry migration. The predicted supply and demand were computed by classification according to industrial sector using the average standard level for 2002–06. Predicted supply-demand imbalance of industry-specific labor force is shown based on predicted worker size, assuming that the fraction of inter-industry migrant from one industry to the other was (A) 0%, (B) 30% and (C) 50%, respectively. Positive value reflects the predicted shortage of experts, i.e., demand minus supply. (TIF 388 kb) [file 12976_2018_91_MOESM5_ESM.tif]

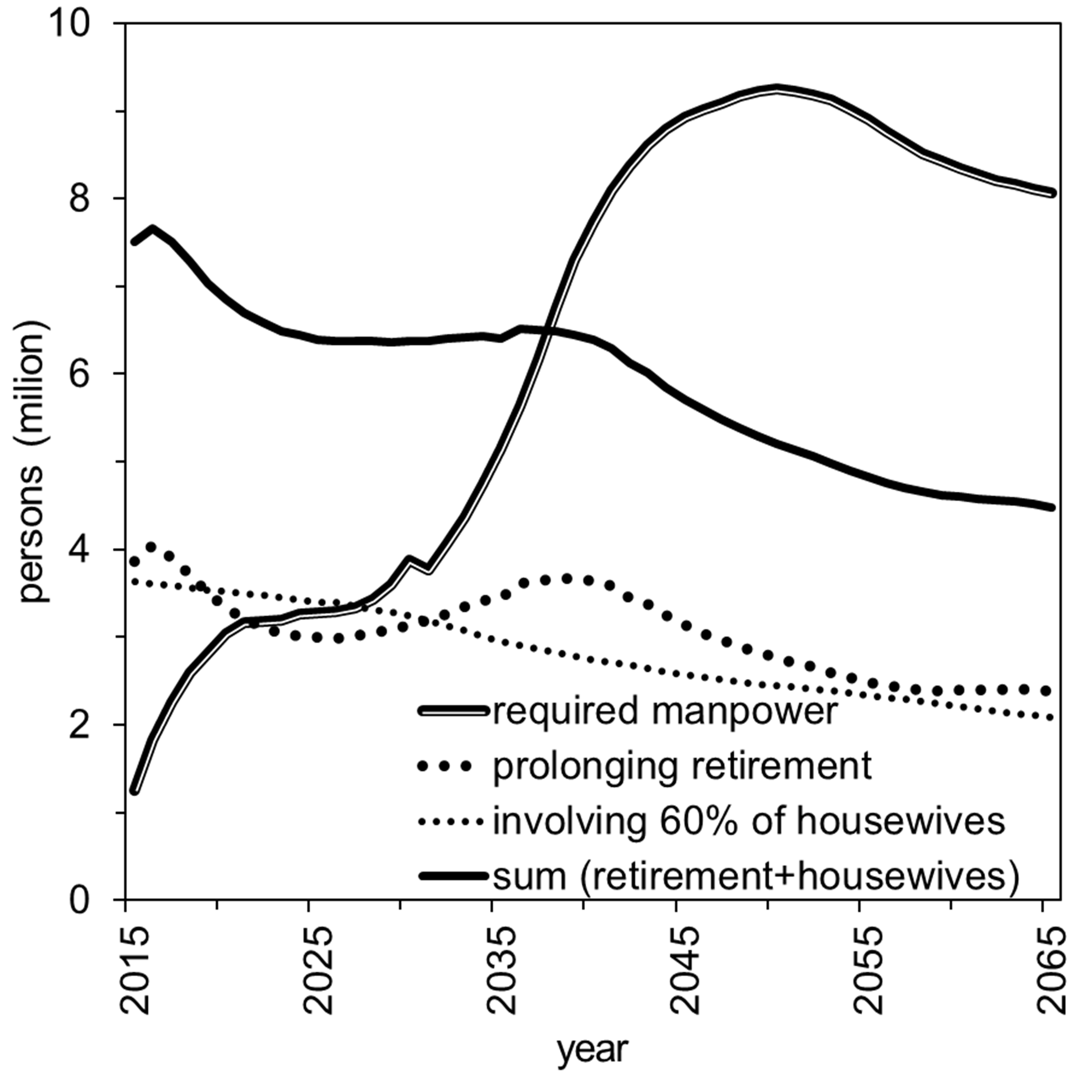

Supplement: Supplementary file 6 — Figure S6. Scenario analysis of the comparison between the expected excess of demand against possible additional number of workers. Deficiency of full-time workers (thick solid line) compared with possible countermeasure scenarios of recruiting 60% of homemakers, extending the retirement age by 10 years, and both options. Even if both countermeasures were undertaken, the shortfall would not be met by 2040. (TIF 568 kb) [file 12976_2018_91_MOESM6_ESM.tif]
